# Electro-mechanical light modulator based on controlling the interaction of light with a metasurface

**DOI:** 10.1038/s41598-017-05906-9

**Published:** 2017-07-14

**Authors:** Pablo Cencillo-Abad, Jun-Yu Ou, Eric Plum, Nikolay I. Zheludev

**Affiliations:** 1 Optoelectronics Research Centre and Centre for Photonic Metamaterials, University of Southampton, Highfield, Southampton, SO17 1BJ UK; 2 Centre for Disruptive Photonic Technologies, School of Physical and Mathematical Sciences and The Photonics Institute, Nanyang Technological University, Singapore, 637371 Singapore

## Abstract

We demonstrate a reflective light modulator, a dynamic Salisbury screen where modulation of light is achieved by moving a thin metamaterial absorber to control its interaction with the standing wave formed by the incident wave and its reflection on a mirror. Electrostatic actuation of the plasmonic metamaterial absorber’s position leads to a dynamic change of the Salisbury screen’s spectral response and 50% modulation of the reflected light intensity in the near infrared part of the spectrum. The proposed approach can also be used with other metasurfaces to control the changes they impose on the polarization, intensity, phase, spectrum and directional distribution of reflected light.

## Introduction

From their beginnings as microwave frequency selective surfaces^1^, metasurfaces have developed into a diverse branch of nanophotonics^2^. Such essentially planar arrays of resonators of sub-wavelength size are being used as spectral filters, wave plates^3^, polarizers^4, 5^ and — based on spatially varying resonators — for redirection^6–8^ and focusing^9^ of light as well as holography^10, 11^. Dynamic control over such structures has been achieved by modifying the materials that make up a metasurface, e.g. using phase transitions^12–14^ or optical nonlinearities^15, 16^, by nanomechanical rearrangement of the array of coupled resonators^17, 18^, and by controlling the metasurface excitation with counterpropagating coherent beams of light^19^. However, these approaches rely on special materials and operating conditions (e.g. temperature, intensity); or complex and fragile nanostructures; or extremely stable interferometric setups.

Here we use nanoelectromechanical actuation to control metasurface excitation in a robust way that is compatible with ambient conditions. We place a plasmonic metasurface absorber of nanoscale thickness a few microns in front of a mirror. Using electrostatic forces, we move the metasurface between positions of constructive interference and positions of destructive interference to control the light-metasurface interaction, resulting in high-contrast modulation of absorption and significant spectral shifts of the structure’s absorption resonances. The combination of a metasurface with a backing mirror is known as Salisbury screen^1, 20, 21^ and has been used previously to control optomechanical resonances^22^, to realize a mid-infrared electro-optical switch^23^ and to enhance metasurface performance, e.g. to achieve phase gradient metasurfaces with high efficiency^24, 25^ as well as handedness-maintaining^26^ and handedness-inverting^27^ mirrors for a single circular polarization. Here we highlight that dynamic control over the spacing of a metasurface of substantially sub-wavelength thickness and a backing mirror provides an effective solution for tuning, modulating and switching the various optical functionalities metasurfaces can provide.

Any material of substantially sub-wavelength thickness may be placed at a node or anti-node of a standing wave formed by coherent counterpropagating waves. At an electric field node of the standing wave, there is no electric field that could interact with the material and therefore the electric light-matter interaction vanishes. In contrast, at an electric field anti-node, the electric field amplitude doubles due to constructive interference, leading to enhancement of the electric light-matter interaction. Thus, the light-matter interaction may be controlled by modulating the relative position of standing wave and material. Such coherent control of light with light has been exploited to control absorption of light from almost 0% to almost 100%^19^, to control the propagation direction of light interacting with phase gradient metasurfaces^28^ and to control polarization of light interacting with anisotropic metasurfaces^29^, with applications including all-optical logic gates^30^ and parallel information processing^31^. These works have been based on meter-scale laser Mach-Zhender interferometers, which had to be stable on a length-scale of few nanometers as nodes and anti-nodes are separated by a quarter of the wavelength. Our approach avoids such challenges by shrinking the interferometer size by 5–6 orders of magnitude. By placing the metasurface in the standing wave that forms in front of a mirror we create a Fabry-Perot microcavity of variable length that controls the light-metasurface interaction.

## Results and Discussion

**Figure 1:**
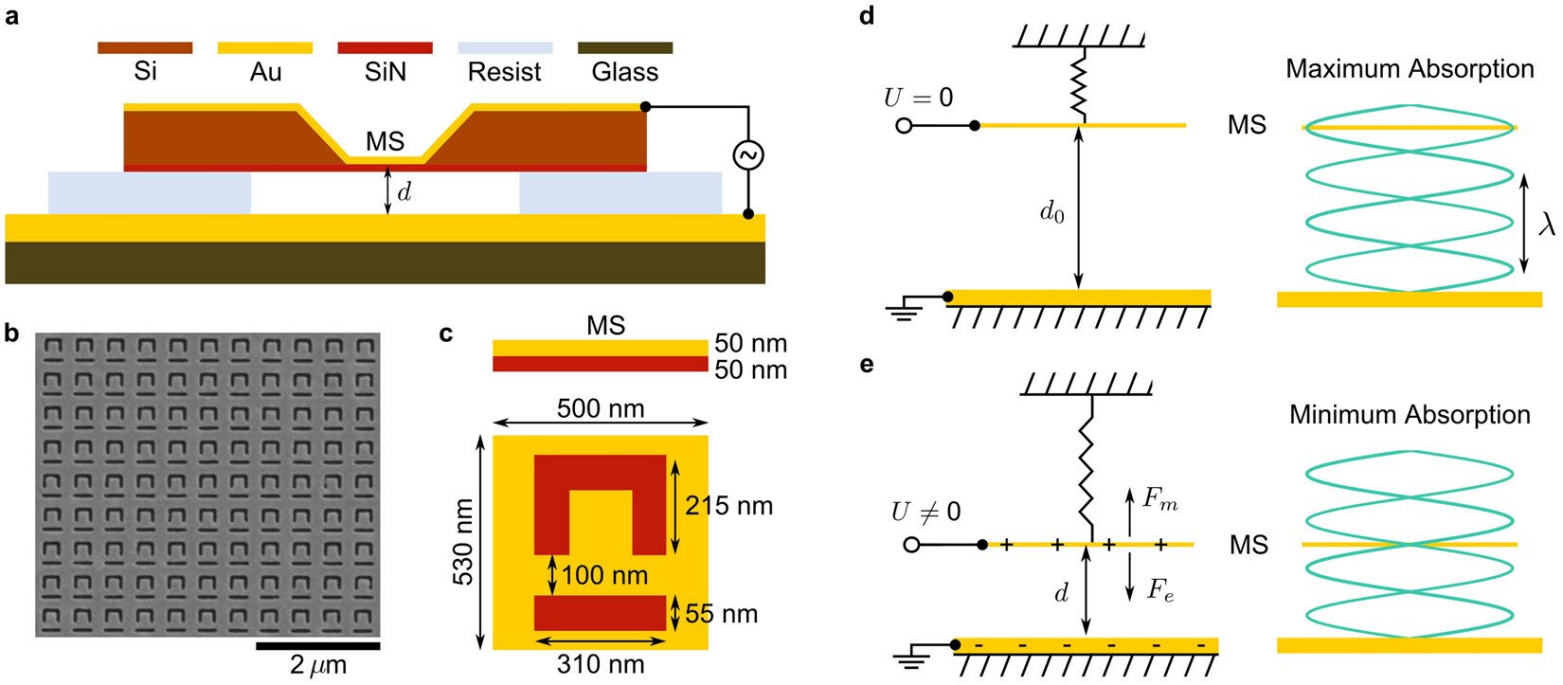
Electrostatic control of metasurface excitation. (**a**) Schematic cross-section of the metadevice consisting of a nanostructured gold-coated silicon nitride membrane separated from a gold-coated glass substrate by a resist spacer layer. (**b**) Scanning electron micrograph of a fragment of the lossy metasurface (MS) consisting of asymmetrically split ring apertures in 50 nm of gold on 50 nm of silicon nitride and (**c**) its cross-section and unit cell. (**d**,**e**) Operating principle of the device that is actuated by application of a voltage *U* leading to an electrostatic force *F*_*e*_ balanced by an elastic restoring force *F*_*m*_. The resulting change of the metasurface-to-mirror distance *d* translates the metasurface between positions of (**d**) constructive interference corresponding to enhanced light-metasurface interaction and (**e**) destructive interference corresponding to suppressed light-metasurface interaction.

The metadevice consists of an electrostatically actuated metasurface that forms a Fabry-Perot microcavity of variable length with a gold mirror as illustrated by Fig. 1. The metasurface is an array of asymmetrically split ring apertures in a 50-nm-thick gold layer supported by a 50-nm-thick silicon nitride membrane, see Methods for details. Asymmetrically split ring apertures were chosen as they are well-studied^32^ and known to provide coherent perfect absorption^19^. Application of an electrical voltage *U* to the gold layers of the device leads to charge accumulation in the elastic metamaterial and the gold mirror in a way that is similar to a parallel plate capacitor. The resulting electrostatic force *F*_*e*_ bends the nanomembrane metamaterial to an equilibrium point where it is balanced by the mechanical restoring force *F*_*m*_ of the elastic structure. Thus, the applied voltage *U* controls the metasurface-to-mirror distance *d* (cavity length). We note that the electrostatic force grows infinite as the distance approaches zero, while the elastic force scales linearly with the deformation, leading to unstable behaviour for sufficiently large *U*. Indeed, a pull-in voltage *U*_*P*_ can be defined above which the electrostatic force cannot be compensated by elastic forces resulting in membrane-to-mirror stiction and device failure. Following^33^, the relationship between applied voltage and resulting metamaterial displacement Δ*d* = *d*_0_ − *d* (cavity length change) is given by

1 $${U}^{2}=\frac{27}{4}\frac{{\rm{\Delta }}d}{{d}_{0}}{(1-\frac{{\rm{\Delta }}d}{{d}_{0}})}^{2}{U}_{P}^{2},$$

where *d*_0_ is the metasurface-to-mirror distance without applied voltage and ${\rm{\Delta }}{d}_{P}=\tfrac{1}{3}{d}_{0}$ corresponds to the metasurface displacement at the pull-in voltage, i.e. the displacement at which device failure is expected. For small displacements, ${\rm{\Delta }}d\ll {d}_{0}$, the displacement is proportional to the square of the applied voltage,

2 $${\rm{\Delta }}d\simeq \frac{4}{27}\frac{{U}^{2}}{{U}_{P}^{2}}{d}_{0}.$$

The optical properties of the metadevice are most easily understood by considering the standing wave that forms in front of a mirror. For a given wavelength of light, the metasurface may be placed at an anti-node or node of the standing wave, depending on the voltage applied to the metadevice, see Fig. 1d,e. At an electric field anti-node, light will interact strongly with the lossy metasurface resulting in large absorption that can reach 100% (coherent perfect absorption). In contrast, absence of electric field at a node will lead to weak light-metasurface interaction with 0% absorption in the ideal case (coherent perfect transparency).

An accurate description of the metadevice’s optical properties must consider the characteristics of the Fabry-Perot cavity formed by metasurface and mirror as well as the optical response of the metasurface itself. Throughout this work we consider linearly polarized light with the electric field oriented parallel to the symmetry axis of the metamaterial pattern.

The optical response of a Fabry-Perot cavity formed by a partially transparent interface and a mirror is determined by multiple reflections of light and its absorption *A* and reflectivity *R* are given by

3 $$A(\lambda )=1-R(\lambda )=1-{|{r}_{{\rm{o}}}+\frac{m{t}^{2}{e}^{-i4\pi d/\lambda }}{1-m{r}_{{\rm{i}}}{e}^{-i4\pi d/\lambda }}|}^{2}$$

where *r*_o_(*λ*) and *r*_i_(*λ*) are the complex Fresnel reflection coefficients for illumination of the partially transparent interface from outside and inside the cavity, *t*(*λ*) is its transmission coefficient, $m(\lambda )\simeq -1$ is the mirror’s reflection coefficient and 2*πd*/*λ* is the phase accumulated during propagation of the wave of wavelength *λ* from interface to mirror^34^. In the cases considered here, absorption by the mirror is small and absorption of the cavity is therefore dominated by absorption in the partially transparent interface, which is controlled by constructive/destructive interference of incident and multiply reflected waves on the interface.

While equation (3) implies interference effects for any cavity length, observation of the cavity resonances with good visibility requires the cavity length *d* to be small compared to the coherence length ${l}_{c}\simeq c/{\rm{\Delta }}f$, where *c* is the speed of light and Δ*f* is the spectral bandwidth in terms of frequency. For larger cavity lengths, interference effects average out over the detected spectral band which will contain wavelengths experiencing both constructive and destructive interference on the lossy interface. Notably, for a narrow-band light source detected with a broadband detector Δ*f* is the spectral bandwidth of the light source, while for a broadband light source detected with a narrowband detector (or a spectrometer) Δ*f* is the spectral bandwidth (or resolution) of the detector. Assuming linear superposition of light at different wavelengths, this effect of temporal coherence on the measured absorption *A*_M_ can be described by

4 $${A}_{{\rm{M}}}=\int P(\lambda )A(\lambda )d\lambda ,$$

where *P*(*λ*) is the normalized product of spectral power distribution and detector sensitivity. For measurements with a spectrometer this applies to each spectral bin and for our experimental wavelength range of 950 nm–2000 nm with a spectral resolution of 7 nm the coherence length ranges from 136 *μ*m to 286 *μ*m.

**Figure 2:**
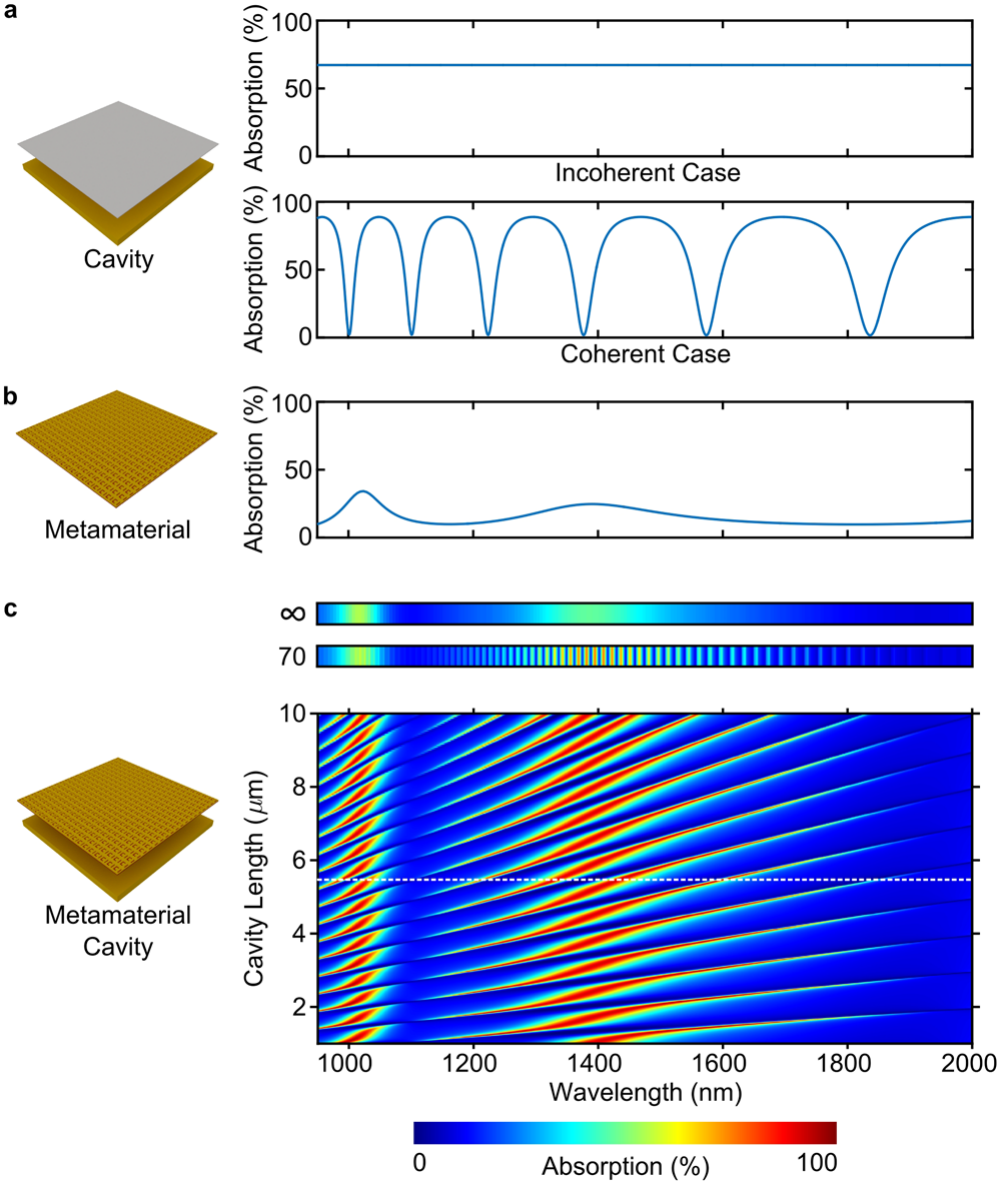
Interactions between Fabry-Perot cavity and metasurface. (**a**) Absorption of a Fabry-Perot cavity formed by a gold mirror and an ideal lossy beam splitter of infinitesimal thickness in the limiting cases corresponding to cavity lengths much larger (top) and much smaller (bottom) than the coherence length. The coherent case assumes a cavity length of *d*_0_ = 5485 nm. (**b**) Absorption spectrum of the metasurface. (**c**) Absorption spectrum of the metadevice consisting of metasurface and gold mirror as a function of cavity length *d* assuming 7 nm detector bandwidth. The experimental cavity length *d*_0_ = 5485 nm is indicated by a dashed line. All results shown in this figure are simulations.

In order to disentangle the effects of Fabry-Perot cavity and metasurface, we will first consider a Fabry-Perot cavity formed by a gold mirror and an ideal lossy beam splitter in the incoherent and coherent regimes, characterized by the cavity length being much larger and much smaller than the coherence length, respectively. An ideal lossy beam splitter exhibits the largest possible absorption for planar metasurfaces, which is 50% of a single beam illuminating one side of the metasurface^35^, and its Fresnel reflection and transmission coefficients are *r*_o,i_ = −0.5 and *t* = +0.5. As illustrated by Fig. 2a, in the coherent case, the Fabry-Perot cavity exhibits alternating absorption maxima and minima, corresponding to constructive and destructive interference of light on the lossy beam splitter, respectively, while no interference effects can be seen in the incoherent case. Figure 2b shows the simulated absorption spectrum of the metasurface, which has absorption resonances at wavelengths of about 1024 nm and 1389 nm, see Methods for details on numerical modelling. Figure 2c shows the absorption spectrum of the whole metadevice consisting of metasurface and mirror as a function of the cavity length *d*. For cavity lengths that are small compared to the coherence length, the absorption spectrum of the metadevice corresponds to Fabry-Perot resonances with an envelope that resembles the absorption spectrum of the metamaterial. Negligible light-metasurface interaction due to destructive interference of light on the metasurface when the cavity length is a multiple of *λ*/2 is apparent as absorption minima corresponding to dark blue straight lines on the colour map. Indeed, for any ideal planar metasurface (*t* = *r*_o,i_ + 1) and an ideal mirror (*m* = −1) absorption according to equation (3) is zero in this case. In contrast to the absorption minima, both spectral position and amplitude of the absorption maxima depend on the optical properties of the metasurface in addition to the cavity length. As the achievable maximum displacement Δ*d*_*p*_ is proportional to the cavity length, longer cavities enable a larger dynamic range of absorption modulation, provided that the cavity length remains small compared to the coherence length. However, as the voltage required to achieve a given displacement is also proportional to the cavity length, longer cavities imply larger actuation voltages. The spectral separation of the Fabry-Perot resonances decreases with increasing cavity length, preventing their detection when the cavity length becomes half of the coherence length as their spectral separation equals the spectral resolution of the spectrometer in this case. For our spectral resolution of 7 nm, the coherence length is 140 *μ*m at about 1000 nm wavelength, causing the Fabry-Perot resonances at this wavelength to vanish for a cavity length of 70 *μ*m, while Fabry-Perot resonances at longer wavelengths remain visible as they correspond to longer coherence lengths, see Fig. 2c. For large cavity lengths, the Fabry-Perot resonances cannot be resolved resulting in a metadevice absorption spectrum that is similar to that of the metasurface.

**Figure 3:**
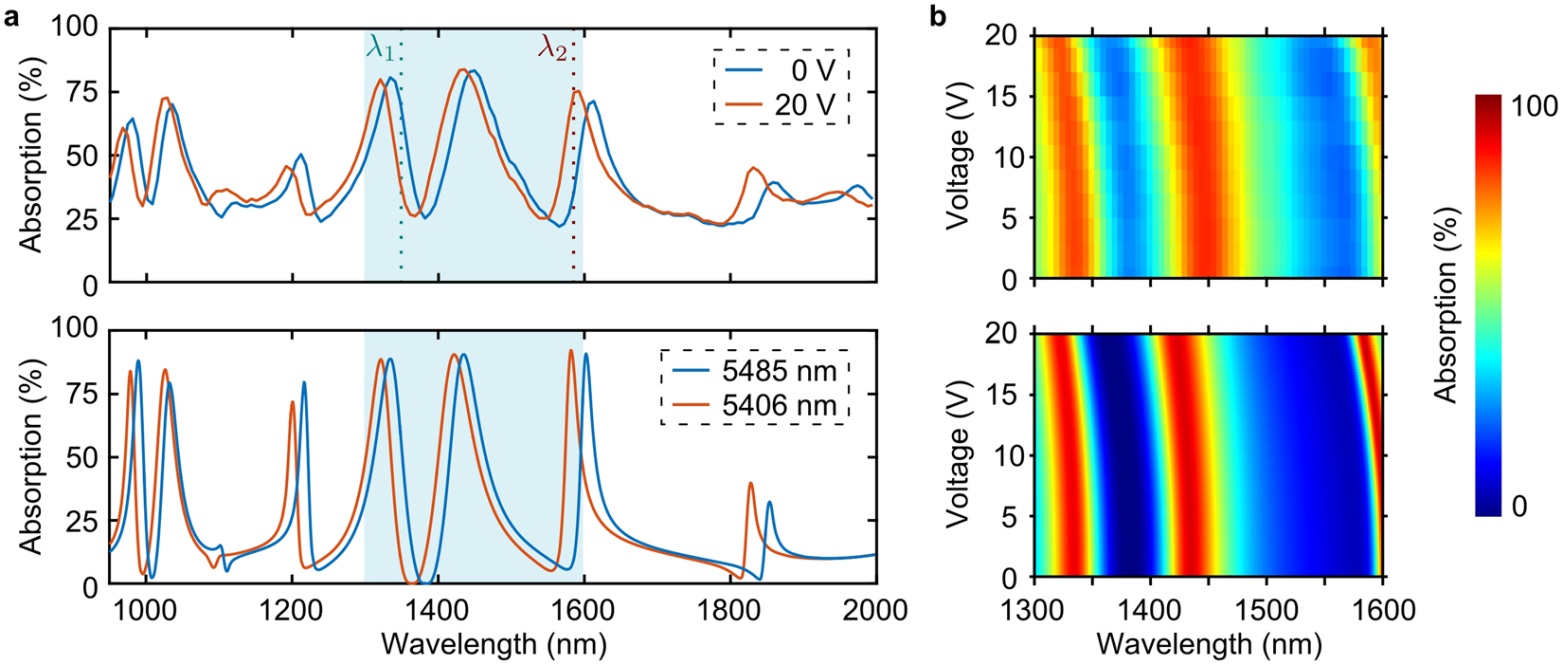
Electric tuning of absorption bands. (**a**) Absorption spectra measured with voltages *U* of 0 V and 20 V applied to the metadevice (top) and best fit theoretical absorption corresponding to metasurface-to-mirror spacings *d* of 5485 nm and 5406 nm based on equation (4) assuming the experimental detector bandwidth of 7 nm (bottom). (**b**) Experimental and theoretical absorption spectra as a function of voltage *U*, where theoretical spectra are calculated as above for cavity lengths *d* = *d*_0_ − Δ*d* given by equation (1) with *U*_*P*_ = 66 *V* and *d*_0_ = 5485 nm.

**Figure 4:**
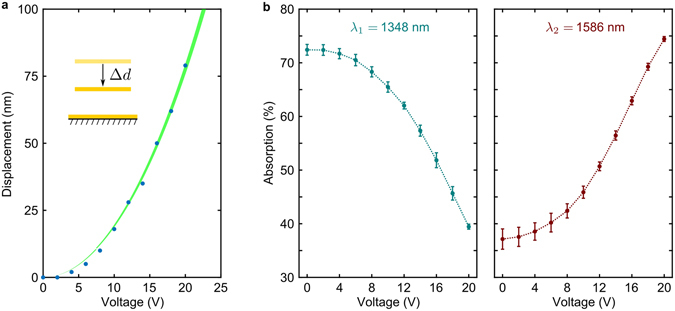
Electric control over metadevice deformation and absorption. (**a**) Metasurface displacement Δ*d* as a function of voltage *U*, where the data points were obtained by fitting the experimental spectra as in Fig. 3a and the green curve corresponds to equation (1) with *U*_*P*_ = 66 *V* and *d*_0_ = 5485 nm, with line width indicating the 95% confidence interval of the fit. (**b**) Tuning of absorption at *λ*_1_ = 1348 nm and *λ*_2_ = 1586 nm by gradual voltage application, where dotted lines serve as an eye guide.

Figure 3a shows absorption spectra of the metadevice measured with 7 nm spectral resolution for applied voltages of 0 V and 20 V (see Methods) alongside numerical fits based on equation (4), where the cavity length *d* is the only fitting parameter. The spectra show absorption maxima and minima corresponding to enhanced and suppressed metasurface excitation due to constructive and destructive interference, respectively. The largest absorption is found near the metasurface resonances and the spectral positions of measured and simulated absorption maxima and minima are in close agreement. Gallium implantation during focused ion beam milling of the metasurface, nanofabrication inaccuracies, a small variation of the cavity length across the metasurface area and focused illumination with a 0.28 numerical aperture objective contribute to reduced contrast in the experimental absorption spectra. Application of 20 V reduces the cavity length from *d*_0_ = 5485 nm to 5406 nm, that is by 79 nm. This cavity length change of 1.44% leads to a blue-shift of the metadevice’s spectral response of the same magnitude, i.e. a 20 nm blue-shift around 1400 nm wavelength. The measured and simulated voltage-dependence of absorption is given by Fig. 3b for the spectral range of highest contrast and the corresponding metasurface displacements that were determined by fitting the experimental absorption spectra are shown by Fig. 4a. Both spectral shift and metasurface displacement are proportional to the square of the applied voltage within our experimental range of voltages. The voltage-dependence of the metasurface displacement closely follows equation (1) with a pull-in voltage of *U*_*P*_ = 66 *V* corresponding to a maximum displacement of ${\rm{\Delta }}{d}_{P}\simeq 1.8$ *μ*m. Thus, our cavity length of *d*_0_ = 5485 nm is big enough to allow a large dynamic range of modulation and sufficiently small to operate the metadevice with reasonable actuation voltages within the coherent regime. Throughout our experiments, we limited the applied voltage to a maximum of 20 V, which is well below the pull-in voltage and thus avoids any risk of electrical damage, while being sufficient for metadevice operation and characterization.

The voltage-induced spectral shift of the metadevice absorption spectrum leads to large intensity changes at specific wavelengths, which may be exploited for tuning and modulation. This is illustrated by Fig. 4b for selected wavelengths of *λ*_1_ = 1348 nm and *λ*_2_ = 1586 nm, where absorption and reflection can be changed by a factor of two. Gradual changes of the applied voltage between 0 V and 20 V lead to gradual changes in absorption. On cycling of the applied voltage, the values were reproduced within 2% in terms of absolute absorption.

**Figure 5:**
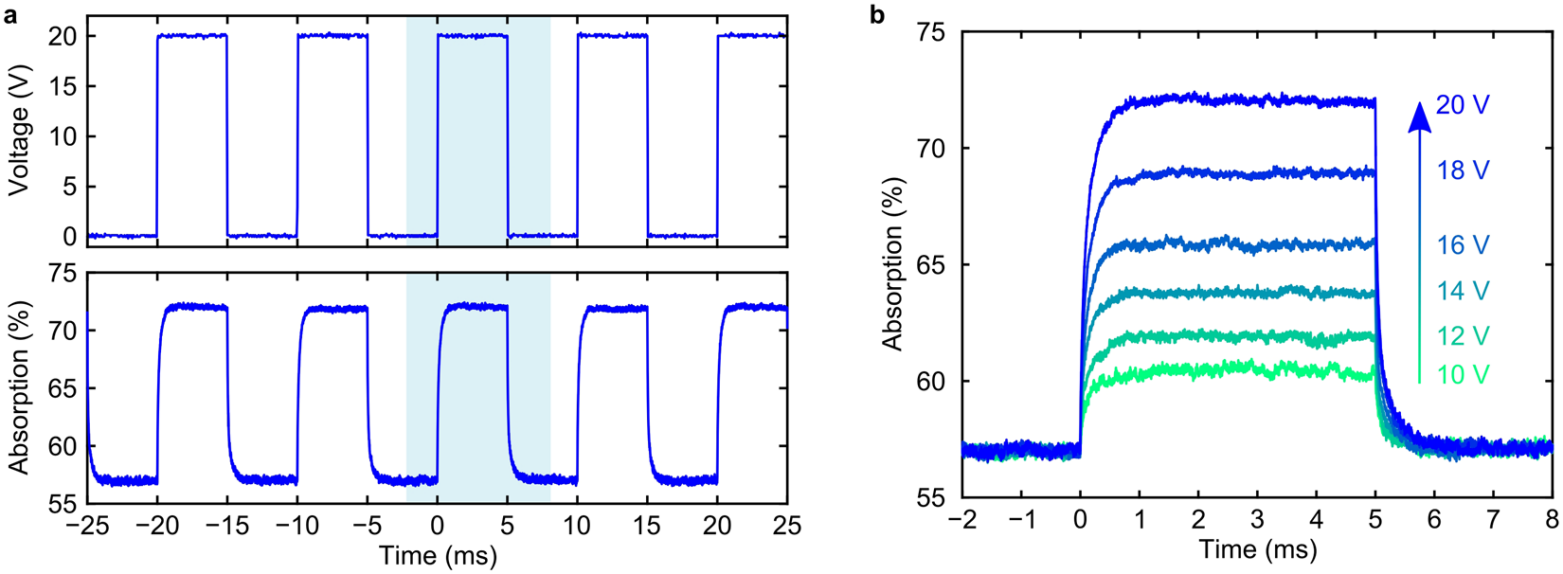
Modulating metasurface absorption. (**a**) Square modulation of the applied voltage *U* between 0 V and a peak voltage of 20 V (top) at 100 Hz and the resulting absorption modulation at a wavelength of 1310 nm (bottom). (**b**) One cycle of modulation for peak voltages from 10 V to 20 V in steps of 2 V.

The dynamic switching behaviour of the metadevice was measured by detecting the structure’s reflectivity at 1310 nm wavelength while modulating the actuation voltage (see Methods). Figure 5 shows how the metadevice absorption changes in response to application of a step-like voltage. Upon voltage application and withdrawal, the absorption — and thus the metasurface-to-mirror spacing — approaches its new equilibrium position exponentially with a characteristic time constant of about 150 *μ*s. Thus, the switching behaviour of the metadevice is consistent with an overdamped harmonic oscillator, where the air surrounding the metadevice is a source of damping. In general, the temporal resolution of nanomembrane metamaterials is controlled by the dimensions of the metasurface membrane, the density and Young’s modulus of the constituent materials and the damping effect of the surrounding atmosphere^17^. Faster modulation can be achieved by operating a metadevice based on a smaller membrane in a low pressure environment. Like metasurface displacement and slow tuning of metadevice absorption, as shown in Fig. 4, also the magnitude of the observed absorption modulation is approximately proportional to the square of the applied voltage.

The optical damage threshold of the metadevice due to melting of the gold nanostructure is controlled by heat transport away from the metasurface by thermal conduction along the metasurface membrane and convection by the surrounding atmosphere. We did not observe any thermal issues in our experiments conducted at sub-milliwatt power levels, however, based on the thermal properties of similar plasmonic nanomembrane metamaterials^36^ we expect that our metadevice can tolerate illumination with at least several milliwatts.

Absorption of our metadevice is a measure of metasurface excitation. Therefore, we argue that our method can be employed to control the light-matter interaction of any metasurface or functional film of substantially sub-wavelength thickness in a reliable and repeatable way at ambient conditions. For example, phase gradient metasurfaces for holography, redirection and focusing of light may be turned on and off, while anisotropic and chiral metasurfaces may become polarization modulators. Metasurfaces or thin films can also be separated into smaller elastic sections with individual electrical contacts, e.g. strips, in order to achieve dynamic spatial control over their functionality﻿^37^. It has been demonstrated numerically, that reconfigurable elements of sub-wavelength spacing can deliver effectively continuous spatial control over the intensity of light^34^.

## Conclusion

In summary, we demonstrate dynamic control over metasurface excitation by assembling a Fabry-Perot microcavity from a gold mirror and an elastic metasurface that is actuated by electrostatic forces. The metadevice acts as an electrically controlled spectral filter and intensity modulator achieving an intensity contrast of two in our experiments. While our metadevice modulates absorption of light, we argue that our method may be used to control any of the diverse optical functionalities that metasurfaces and films of substantially sub-wavelength thickness can provide.

## Methods

### Metadevice fabrication

The metadevice was fabricated starting with a commercially available 500 × 500 *μ*m^2^ silicon nitride membrane of 50 nm thickness that is supported by a 5 × 5 mm^2^ silicon frame (Norcada Inc) and a glass substrate, see Fig. 1. The mirror was fabricated by coating the glass substrate with 200 nm of gold using thermal evaporation. Using the same technique, the membrane was coated with a 50-nm-thick gold layer that was then perforated by focused ion beam milling to create the metasurface, an array of asymmetrically split ring apertures. The metasurface has an overall size of 30 × 30 *μ*m^2^ with a 500 × 530 nm^2^ unit cell. Photolithography resist (S1813) was used as a spacer on top of the gold mirror. A central groove of about 1 mm width was exposed and developed to generate the gap of the resonant cavity. The metadevice was finally assembled by placing the membrane in front of the mirror, taking care to align the groove with the membrane window.

### Simulations

Numerical modelling uses a Drude-Lorentz model with 3 oscillators for the electric permittivity of gold^38^ and a constant permittivity of 4.0 for silicon nitride. The optical properties of the metasurface were simulated for normal incidence illumination by a plane wave considering a single unit cell with periodic boundary conditions using finite element method modelling (COMSOL Multiphysics 4.4) in three dimensions.

### Experimental metadevice characterization

Absorption spectra of the metadevice — which cannot transmit light — were determined from its reflectivity spectra that were measured with applied voltages between 0 V and 20 V using a microspectrophotometer (CRAIC Technologies) with a halogen light source and 7 nm spectral resolution, see Figs 3 and 4b. The dynamic switching behaviour of the metadevice was measured by detecting the reflection of a 1310 nm CW laser using a photodetector and an oscilloscope, while modulating the actuation voltage with a function generator, see Fig. 5.

### Data Availability

The data from this paper can be obtained from the University of Southampton ePrints research repository: http://doi.org/10.5258/SOTON/D0044.
